# The Development of an Extraction Method for Simultaneously Analyzing Fatty Acids in Macroalgae Using SPE with Derivatization for LC–MS/MS

**DOI:** 10.3390/molecules29020430

**Published:** 2024-01-16

**Authors:** Taewoo Yum, Eun-Yong Kim, Yeongeun Kim, Sukyoung Choi, Ki-Jung Paeng

**Affiliations:** Department of Chemistry, Yonsei University, Wonju 26493, Republic of Korea; t7630@naver.com (T.Y.); ssatang@yonsei.ac.kr (E.-Y.K.); kyewow1984@naver.com (Y.K.); 01041712@naver.com (S.C.)

**Keywords:** macroalgae, fatty acid, trimethylaminoethyl derivatives, solid-phase extraction, LC–ESI–MS/MS

## Abstract

Fatty acid analysis is an essential step in evaluating the potential of macroalgae for biodiesel production. An extraction method was developed to simultaneously analyze up to five types of biodiesel-fuel-related fatty acids (myristic acid, palmitic acid, *cis*-palmitvaccenic acid, stearic acid, and oleic acid) in macroalgae using liquid chromatography and tandem mass spectrometry (LC–MS/MS). Lypophilization and solid-phase extraction (SPE) techniques were applied to improve the extraction efficiency and effectively purify samples. The optimal conditions for SPE were set by comparing the recoveries according to the various solvent conditions for each step (loading, washing, and elution). In addition, the introduction of trimethylaminoethyl (TMAE) derivatives to the hydroxyl group of the target analyte increased the ionization efficiency and sensitivity. The derivatized samples were analyzed using the LC–MS/MS method with electrospray ionization in the positive and multiple-reaction monitoring modes. The target analytes were separated and detected within 13.5 min using a CAPCELL PAK C18 MGII S3 column. Gradient elution was performed using distilled water and acetonitrile containing 5 mM ammonium acetate. This method offers a reliable and sensitive tool for the analysis of macroalgae samples for their potential use in biodiesel production. To the best of our knowledge, this is the first report on the simultaneous determination of fatty acids in macroalgae using LC–MS/MS with TMAE derivatization.

## 1. Introduction

Biodiesel fuel is a renewable and environmentally friendly alternative to fossil fuels owing to its renewability, sustainability, and carbon neutrality [1,2,3,4]. Biodiesel can be divided into three main categories: solid, liquid, and gas. Liquid biofuels are attracting attention and are being studied because of their high use-value as a major fuel for internal combustion engines. Among the various biofuels that have been tested, algae are one of the most promising energy sources [5]. They offer fast growth, high yields, the adaptability of living in a variety of environments, the vastness of arable land, and many competitive advantages over ground energy sources [6,7,8,9]. Also, they are promising sources of oil for biodiesel production owing to their high fatty acid contents [10,11].

Algae can be classified as micro or macro, depending on their size [12]. Macroalgae, also known as seaweed, are multicellular plants, making their harvest easier than that of microalgae [13]. Macroalgae are composed of various species, and the most common fatty acids contained in macroalgae are palmitic acid (PA, 16:0), oleic acid (OA, 18:1), α-linolenic acid (18:3), arachidonic acid (20:4), and eicosapentaenoic acid (20:5). *Phaeophyceae* (brown algae) have a high content of saturated fatty acids, including myristic acid (MA, 14:0) and PA. As a result of the analysis of the content of fatty acids in five species (*Porphyra dioica*, *Ceramium virgatum*, *Chondrus crispus*, *Gracilaria gracilis*, and *Palmaria palmata*) of *Rhodophyta* (red algae), PA, *cis*-palmitvaccenic acid (*cis*-PA, 16:1), and oleic acid (OA, 18:1) were detected in high proportions. PA and OA were detected at high abundance in two species (*Codium fragile* and *Ulva lactuca*) of *Chlorophyceae* (green algae) [14]. In a study analyzing fatty acids in nine macroalgal species (*Ulva lactuca*, *Chondrus crispus*, *Laminaria hyperborean*, *Fucus serratus*, *Undaria pinnatifida*, *Palmaria palmata*, *Caulerpa taxifolia*, *Ascophyllum nodosum*, and *Sargassum natans*) from the North Atlantic and tropical seas, MA, PA, *cis*-PA, OA, stearic acid (SA, 18:0), and OA were detected in all the species, and PA especially showed the highest content [15]. Research on tropical green seaweeds (Caulerpa lentillifera and Ulva reticulata) also shows that PA has the highest content, and cis-PA, SA, and OA are included [16]. High fatty acid contents are present not only in macroalgae but also in microalgae. It has been reported that MA, PA, OA, linoleic acid (18:2), and α-linoleic acid (18:3) are synthesized in all seven fresh water microalgae species (*Botryococcus braunii*, *Dunaliella bardawil*, *Dunaliella salina*, *Ankistrodesmus* sp., *Isochrysis* sp., *Nannochloris* sp., and *Nitzschia* sp.). The composition of fatty acids produced by algae varies depending on the species of algae, cultivation conditions, environment, and influencing factors [8,17,18]. Therefore, in this study, we selected five types of fatty acids (MA, PA, cis-PA, SA, and OA) that would mainly be included in macroalgae, considering various algae species and various environments.

Fatty acid analysis is an essential step in evaluating the potential of macroalgae for biodiesel production. Fatty acids are actively analyzed using gas chromatography (GC) and gas chromatography–mass spectrometry (GC–MS) [19,20,21,22,23]. In addition, there are many papers on the analysis of the fatty acid contents in algae using GC and GC–MS [24,25,26]. However, GC and GC–MS have a problem in that the vaporization efficiency of the analyte decreases as the molecular weight of fatty acids increases. The direct analysis of fatty acids in macroalgae using liquid chromatography–mass spectrometry (LC–MS) and liquid chromatography and tandem mass spectrometry (LC–MS/MS) is challenging due to the low ionization efficiency of fatty acids. For the quantitative analysis of fatty acids using LC–MS/MS, target analytes must be sufficiently ionized, and the proper collision energy must be established to generate fragment ions used for quantification. Fatty acids usually exhibit insufficient sensitivity because of their low ionization efficiency [27]. Therefore, a derivatization step should be applied to overcome such problems [28]. The existing algae extraction method is complex, requires multiple steps, and has problems with poor extraction reproducibility. Derivatization with trimethylaminoethyl (TAME) is expected to simplify the extraction step and improve the algae recoveries and extraction reproducibility. In addition, the analytical procedure used for fatty acids in macroalgae analysis is more complex because the matrix samples are very diverse [5]. Fatty acid analysis should be accompanied by additional steps, such as lyophilization, cutting (or mechanical pulverization), co-liquefaction, and solvent extraction (or incubation), to extract target analytes from the carbohydrate matrix. Also, to extract the lipid content from macroalgae, a Soxhlet apparatus is used, or subcritical water extraction is performed. [5,13]. Therefore, removing matrix interferences and selectively extracting analytes are essential in fatty acid analysis, and various methods are applied depending on the characteristics of the analyte and sample matrix. Previous studies on fatty acids from macroalgae have used LC–MS/MS with dilution, LLE, SPE, and matrix elimination as sample preparation methods [11]. However, as far as we know, no studies for simultaneously analyzing MA, PA, *cis*-PA, SA, and OA have been reported to date.

In this study, a TMAE-derivatization-based extraction method was developed for the simultaneous analysis of five types of biodiesel-fuel-related fatty acids (MA, PA, *cis*-PA, SA, and OA) in macroalgae with LC-MS/MS. The introduced TMAE derivatization reaction conditions were fully optimized to overcome the low ionization efficiency of the target analytes. In addition, the extraction efficiencies of the solid-phase extraction (SPE) were compared under various conditions to enhance the sensitivities of the target analytes. This is the first report on the simultaneous determination of these five biodiesel-fuel-related fatty acids in macroalgae using LC–MS/MS with TMAE derivatization based extraction method. The proposed method offers a reliable and sensitive tool for the analysis of large algae samples for their potential use in biodiesel production.

## 2. Results and Discussion

### 2.1. Derivatization Performance

**Figure 1:**
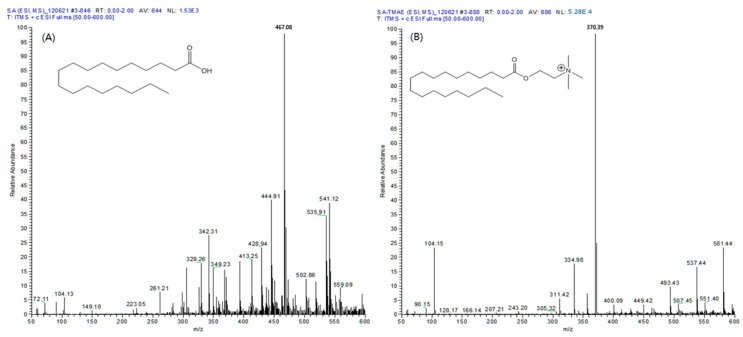
Full scan mass spectrum of (**A**) stearic acid and (**B**) TMAE–stearic acid in positive electrospray ionization mode.

Five types of fatty acids (MA, PA, cis-PA, SA, and OA) reacted through all the same mechanisms, and the [M]^+^ ion was observed at an *m*/*z* value of 89, which is the molecular weight of the fatty acid plus the TMAE group. During the MS/MS analysis, the collision energy of MS generates [M-59]^+^ ions from which the trimethylamine group is separated, making useful quantitative analysis possible. As a result of comparing the full mass spectra of the SA and TMAE–SA derived using electrospray ionization (ESI), the [M + H]^+^ ion of SA (*m*/*z* 285) and sodium and potassium adductive ions (*m*/*z* 307 and 323, respectively) were not observed (Figure 1A). However, in the case of TMAE–SA, the [M + H]^+^ ion of TMAE–SA (*m*/*z* 370) was confirmed, and the relative abundance of the ions showed a satisfactory sensitivity (5.28 × 10^4^). The similar results were confirmed for all the analytes. Therefore, TMAE derivatization could secure sufficient sensitivity for the quantification of analytes in LC–MS/MS analysis.

### 2.2. Selection of Ionization Method-ESI vs APCI

ESI and atmospheric-pressure chemical ionization (APCI) were applied to compare the ionization efficiencies and sensitivities for the derivatized analytes. There were no significant differences for MA, PA, *cis*-PA, and OA in both ionization methods, but a distinctively different pattern was observed in the case of SA. When analyzing TMAE–SA with APCI, no molecular ion was observed, while a molecular ion with sufficient sensitivity (5.28 × 10^4^) was identified using ESI. These results indicated the ESI was more efficient than APCI in fatty acid analysis.

### 2.3. Optimization of LC–MS/MS Conditions

The molecular ions of all the analytes and internal standards (ISs) were efficiently ionized using the positive ESI and multiple reaction monitoring (MRM) modes. The use of the MRM mode increased the selectivity of the target ions while minimizing interferences. All the mass spectrometric parameters, including the capillary voltage and collision energy, were fully optimized to improve the ionization efficiency of each analyte and IS. After going through the derivatization step using the 20 μg/mL standard solution of each analyte and IS, the full-scan spectra of each analyte and IS were obtained to determine the *m*/*z* value of the precursor ion. After fixing either the ion spray voltage or capillary voltage by changing another parameter, the conditions under which the peaks of the analyte and IS precursor ions showed the highest intensity were determined. To determine the fragment pattern and collision energy of each chemical substance, the *m*/*z* value of the product ion was determined from the *m*/*z* value of the selected precursor ion in the full-scan spectrum, and the collision energy corresponding to the most optimized *m*/*z* value of the product ion was optimized. The MRM transitions and mass spectrometric parameters for the analytes and ISs are summarized in Table 1.

**Figure 2:**
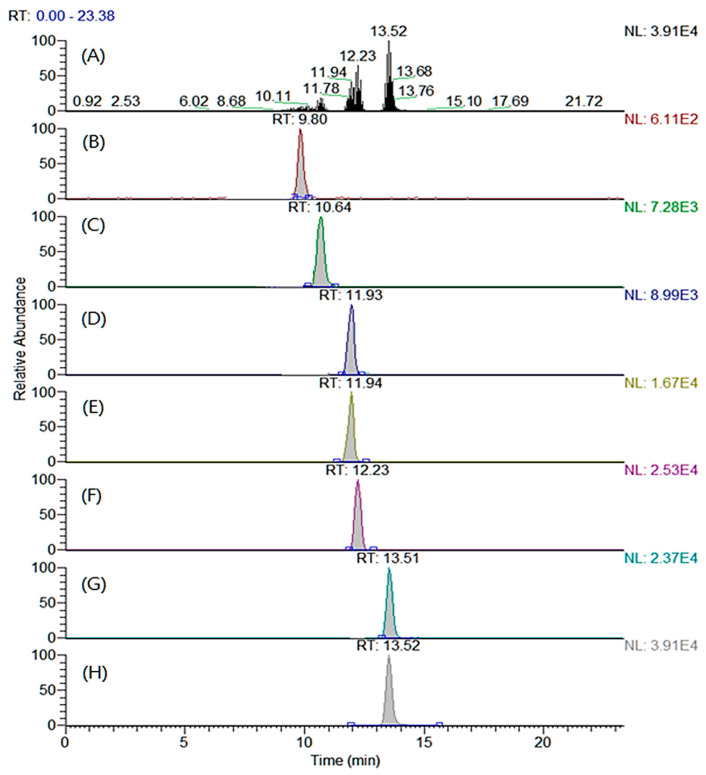
(**A**) Total ion chromatogram and multiple reaction monitoring chromatograms of (**B**) trimethylaminoethyl (TMAE)–myristic acid, (**C**) TMAE–*cis*-palmitvaccenic acid, (**D**) TMAE–palmitic acid, (**E**) TMAE–palmitic acid-d_2_, (**F**) TMAE–oleic acid, (**G**) TMAE–stearic acid, and (**H**) TMAE–stearic acid-d_2_.

The LC columns and mobile phases were tested to determine the optimal separation conditions that maximized the chromatographic efficiency under the optimized MRM conditions. Four types of reverse-phase columns were compared: Shiseido Capcell PAK C18 MGII S3 (2.0 × 100 mm, 3.0 μm particle size), Thermo Hypersil Gold (2.1 × 100 mm, 5.0 μm particle size), Agilent Eclipse XDB-C18 (2.1 × 100 mm, 5.0 μm particle size), and Agilent Zorbax SB-C18 (2.1 × 100 mm, 3.5 μm particle size). All the examined columns showed comparable chromatographic efficiencies. However, the SHISEIDO CAPCELL PAK C18 MGII S3 column displayed a better peak symmetry than those of the other columns. Various additives (ammonium acetate, acetic acid, ammonium formate, and formic acid) and two organic solvents (methanol and acetonitrile) were combined, and the performances were compared for different compositions. The mobile phase composition was selected to maximize the resolution of the analytes because under most conditions, the peaks corresponding to MA and *cis*-PA and PA and OA overlap. The combination of (A) water and (B) acetonitrile containing 5 mM ammonium acetate exhibited the highest sensitivity for all the analytes. Also, the peak resolutions of the above-mentioned analytes (MA versus *cis*-PA and PA versus OA) were significantly improved at this composition. All the target analytes and ISs were separated and detected within 13.5 min by adjusting the gradient conditions. The total ion and MRM chromatograms of the derivatized analytes and ISs are shown in Figure 2.

The ESI source conditions, such as the ion spray voltage, ion source temperature, sheath gas, and auxiliary gas, were also adjusted and optimized by mixing neat standard solutions of the five analytes (MA, PA, *cis*-PA, SA, and OA) at a flow rate of 10 μL/min and optimizing the mobile phase composition (flow rate: 300 μL/min) using post-column infusion.

### 2.4. Optimization of Trimethylaminoethyl Derivatization Conditions

According to previous reports, fatty acids usually exhibit insufficient sensitivity in LC–MS/MS because of their low ionization efficiency [27,28]. Therefore, a derivatization reaction was introduced to overcome such problems in this study. The TMAE derivatization of fatty acids consists of a three-step reaction in general. The mixed standard solution (20 μg/mL for the analytes and ISs) was used to optimize the specific conditions that maximized the efficiency of TMAE derivatization.

**Figure 3:**
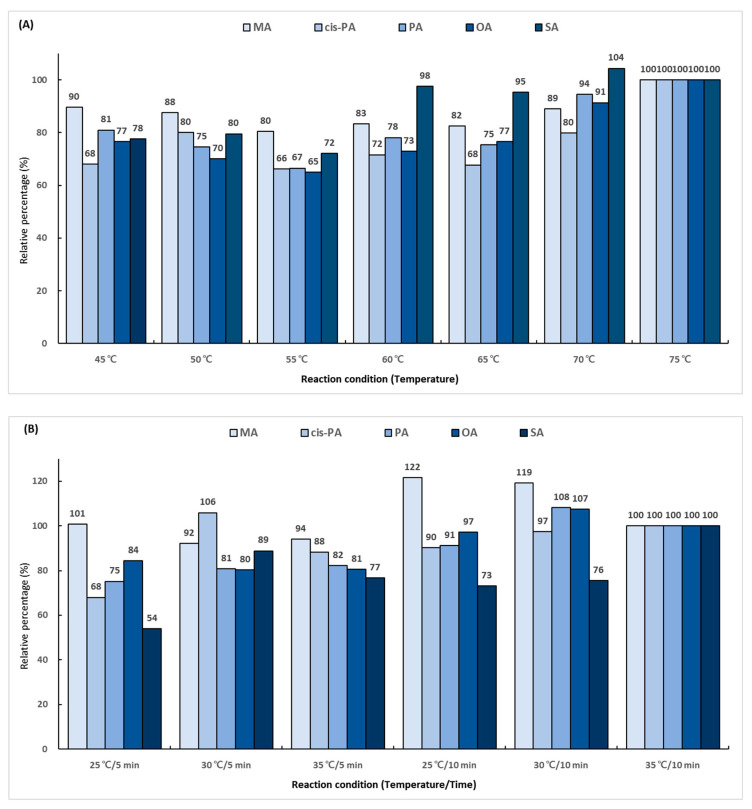

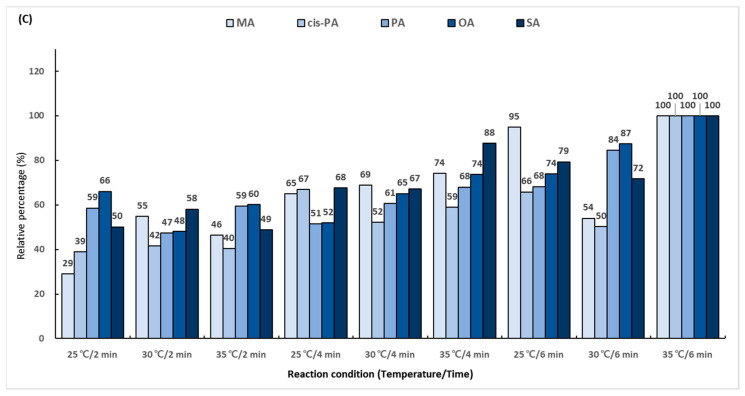
Optimization of trimethylaminoethyl derivatization conditions for (**A**) first reaction using oxalyl chloride, (**B**) second reaction using 2-dimethylaminoethanol, and (**C**) final reaction using iodomethane.

In the first reaction, the hydroxyl group (-OH) in the carboxyl group (-COOH) of the fatty acid is replaced with chlorine (-Cl) using oxalyl chloride. To determine the derivatization efficiency according to the reaction temperature and time, various combinations of the reaction time (5 and 10 min) and reaction temperature (from 45 to 75 °C) were evaluated. A total 14 reaction conditions were employed, and the performance was evaluated as a relative percentage based on the area ratio of the analyte and IS peaks. The derivatization efficiency tended to increase as the reaction temperature was increased (Figure 3A). The conditions of 75 °C and 10 min showed excellent efficiency for all the analytes. The second reaction using 2-dimethylaminoethanol substituted chlorine (-Cl) in the dimethylaminoethyl group. The experiment was conducted using several combinations of the reaction temperature (25, 30, and 35 °C) and reaction time (5 and 10 min). A total of six conditions were employed, and the performance was evaluated as a relative percentage based on the area ratio of the analyte and IS peaks. As a result of comparing the reaction conditions, it was confirmed that the second reaction was the most effective at 35 °C for 10 min (Figure 3B). The final reaction using iodomethane added a methyl group for giving cationic properties to the amino group in the dimethylaminoethyl group. The reaction temperature conditions were set at 25, 30, and 35 °C, and the reaction times were set at 2, 4, and 6 min. A total of nine conditions were employed, and the performance was evaluated as a relative percentage based on the area ratio of the analyte and IS peaks. The highest efficiency was shown at 35 °C for 6 min, which was double the lowest efficiency obtained at 25 °C for 2 min (Figure 3C).

### 2.5. Optimization of Solid-Phase Extraction Conditions

#### 2.5.1. Optimization of Loading Solution

Because macroalgae samples are more complex, the analysis is more complex, and it is necessary to extract target analytes while minimizing any potential matrix interferences. Therefore, the SPE approach was evaluated to eliminate complex interferences released from macroalgae samples and to enhance the peak area of the target analytes compared to baseline noise.

**Figure 4:**
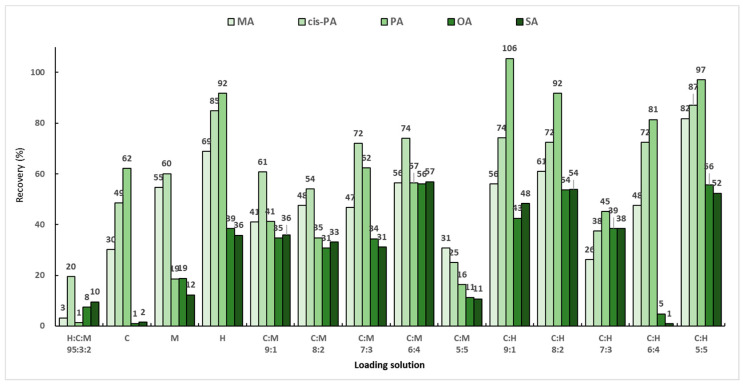
Recoveries according to loading solutions with various solvent compositions.

Background samples and actual samples are powders made by freeze-drying large algae and are insoluble in organic solvents, so a loading solution must first be used to extract the target analyte from the matrix sample to be analyzed. As previously reported, the most efficient loading solution in SPE used to extract fatty acids from muscle foods is a hexane:chloroform:methanol mixed solution (95:3:2, *v*/*v*/*v*) [29]. However, because the target analytes in this study are macroalgae, the composition and efficiency of the loading solution will be different, so the loading solution must be optimized. Three solvents (chloroform, methanol, and hexane) were chosen based on the previous report, and the extraction efficiencies were compared for various loading solutions. To find the optimal loading solution that maximized the extraction efficiency of the analytes, the blank sample was post-spiked with a working standard solution consisting of 200 μL of the mixed standard solution (50 μg/mL) and 200 μL of the mixed IS solution (20 μg/mL). The specific experimental process included the following steps: (1) 200 μL of the analyte (50 μg/mL) was added to a 30 mg background sample; (2) 6 mL of the loading solution was independently added; (3) the mixture was stirred for 1 min; and (4) SPE was applied. Then, each eluate was evaporated and dried under nitrogen gas. The final residue was subjected to an optimized derivative reaction mentioned in Section 2.4. A total of 14 loading-solution compositions were employed, and the performance of the loading solutions was evaluated based on the recoveries of MA, PA, *cis*-PA, SA, and OA. A previously reported mixed solution of hexane:chloroform:methanol (95:3:2, *v*/*v*/*v*) showed the lowest extraction efficiency, while the highest efficiency was observed for the mixed solution of chloroform:hexane (1:1, *v*/*v*). Most of the analytes exhibited sufficient recoveries of more than 80% (Figure 4). However, TMAE–OA and TMAE–SA showed low extraction efficiencies, while only three analytes extracted using chloroform:hexane (1:1, *v*/*v*) exhibited acceptable extraction efficiencies above 50%.

#### 2.5.2. Optimization of Washing Solvent

**Figure 5:**
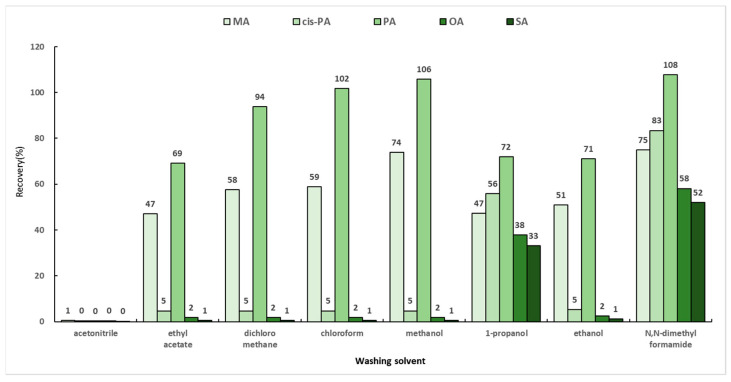
Recoveries by eight washing solvents in solid-phase extraction.

When SPE is applied to samples, various interfering suubstances released from the matrix are stored and maintained in the sorbent along with the target analytes. Therefore, it is important that the target remain within the sorbent while the interferences are removed. Generally, a mixed solution of chloroform and 2-propanol (2:1, *v*/*v*) is used in the washing step of fatty acids [30]. In this study, a total of eight solvents (ethyl acetate, dichloromethane, chloroform, methanol, ethanol, 1-propanol, acetonitrile, and *N*,*N*-dimethylformamide) were selected based on the previous report and their respective polarities. Dichloromethane and ethyl acetate, which have polarities similar to that of chloroform, were selected. In addition, the experiments were conducted by selecting methanol, 1-propanol, and ethanol, which are alcohols like 2-propanol, as washing solvents. When two solvents were mixed, acetonitrile and *N*,*N*-dimethylformamide, which are solvents with similar polarities, were used. The SPE cartridge, which was activated with 3 mL of chloroform and then loaded with 6 mL of the optimized loading solution mentioned in Section 2.5.1 (hexane:chloroform:methanol (95:3:2, *v*/*v*/*v*)), was washed with 4 mL of the washing solvents and eluted with 4 mL of the mixed solution of diethylether and acetic acid (98:2, *v*/*v*). The efficiencies of the washing solvents were compared for the recoveries of MA, PA, *cis*-PA, SA, and OA, and the results are shown in Figure 5. The recoveries by 1-propanol were 33–72%, while *N*,*N*-dimethylformamide showed the highest recovery rates of 52–108%. However, considering the time required for the evaporation step, *N*,*N*-dimethylformamide was not selected as a washing solvent owing to its low volatility. Therefore, the washing solvent of the solid-phase extraction method was chosen as 1-propanol.

#### 2.5.3. Optimized Final Conditions

**Figure 6:**
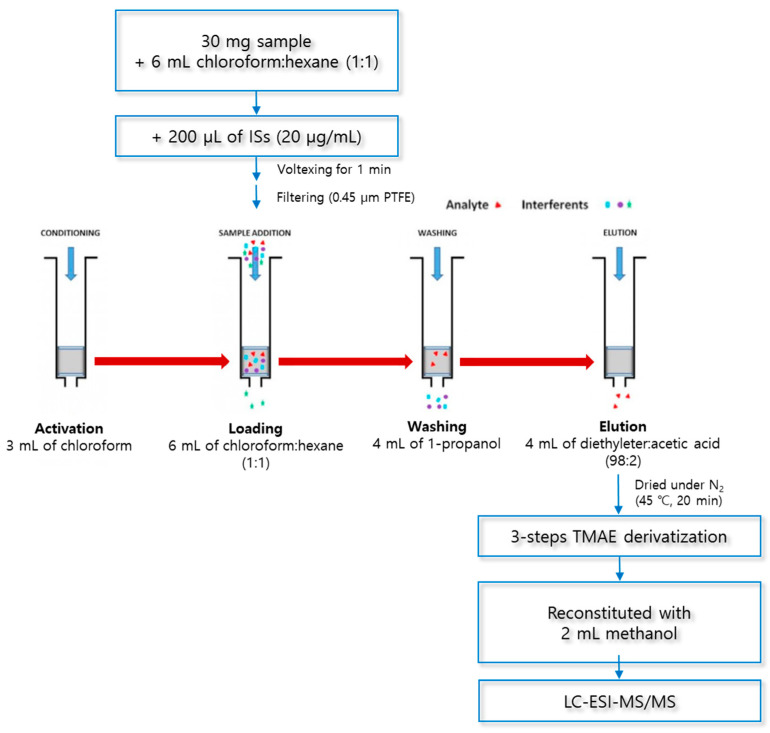
Optimized procedure for solid-phase extraction.

The optimized SPE procedure was as follows (Figure 6): The cartridge was (1) activated with 3 mL of chloroform, (2) loaded with a sample in 6 mL of a chloroform:hexane solution (1:1, *v*/*v*), (3) washed with 4 mL of 1-propahnol, and (4) eluted with 4 mL of a diethylether:acetic acid solution (98:2, *v*/*v*). The dried eluate was derivatized in a three-step reaction, reconstituted with 2 mL of methanol, and analyzed using LC–ESI–MS/MS.

### 2.6. Recovery

The recoveries of MA, PA, cis-PA, SA, and OA were evaluated in samples prepared using the optimized conditions for derivatization and SPE mentioned in Section 2.4 and Section 2.5, respectively. The data of recoveries were obtained using two sets of samples and repeated three times. Set A was the extract of the quality control (QC) sample (pre-extraction sample), and set B was the extract of the blank sample spiked with the same amount of the analyte as in the QC sample after SPE (post-extraction sample). The QC sample was prepared by adding 200 μL of the working standard solution (50 μg/mL) to 30 mg of the blank sample, and both sets underwent the same derivatization process. The recoveries for MA, PA, *cis*-PA, SA, and OA ranged from 80.5 to 103.4%, with corresponding relative standard deviations of <5% RSD. These data were acceptable because they met the recoveries.

## 3. Materials and Methods

### 3.1. Chemicals and Reagents

Fatty acid standards, MA, PA, *cis*-PA, SA, and OA, and ISs, palmitic acid-d_2_ (PA-d_2_) and stearic acid-d_2_ (SA-d_2_), were obtained as solutions from Sigma-Aldrich (St. Louis, MO, USA), AccuStandard (St. Market, NH, USA), and C/D/N Isotope (St. Leacock, QC, Canada). Acetonitrile, methanol, and hexane were purchased from J.T. Baker, Inc. (Phillipsburg, NJ, USA). Ammonium acetate, ethyl acetate, dichloromethane, chloroform, 1-propanol, ethanol, *N*,*N*-dimethylformamide, diethylether, acetic acid, iodomethane, 2-dimethylaminoethanol, and oxalyl chloride were obtained from Sigma-Alrich (St. Louis, MO, USA). All the solvents for the analysis were of HPLC grade or higher. The standard stock solutions of each analyte and IS were prepared using methanol at concentrations of 1 mg/mL. The working standard solutions of the analytes (from 0.2 to 500 µg/mL) and ISs (20 µg/mL) were prepared by appropriately diluting the standard stock solutions with methanol. All the standard solutions were stored at −20 °C in amber bottles before use.

The blank sample contained the species Gigartina tenella (Marine Natural Product 186; MNP 186), which was supplied by the Gangneung branch of the Korea Institute of Science and Technology (Gangneung, Republic of Korea), and an extraction experiment was conducted according to spiking standards.

### 3.2. Sample Preparation (Lyophilization and Solid-Phase Extraction)

Lyophilization was performed for the efficient extraction of fatty acids from macroalgae. Macroalgae samples were washed twice with distilled water. These samples were frozen at −20 °C for 3 h and then dried for 24 h using a freeze-drier (FD-1000, EYELA, Tokyo, Japan). The completely dried samples were made into powders using a pestle and mortar and kept refrigerated at 4 °C until the analysis.

SPE was carried out using aminopropyl columns Mega BE-NH2 (500 mg, 6 mL, Agilent Technologies, Santa Clara, CA, USA) and a 24-port SPE vacuum manifold (Honeywell-Burdick & Jackson, Morris Plains, NJ, USA). To prepare the loading solutions, 30 mg of lyophilized macroalgae was added to 6 mL of a chloroform and hexane mixed solution (1:1, *v*/*v*) and 200 μL of ISs (20 μg/mL). The mixture was vortexed at 1500 rpm for 1 min (Vortex-Genie^®^ 2, Scientific Industries, New York, NY, USA) and filtered using a 0.45 μm PTFE syringe filter (Advantec Co., Tokyo, Japan). Then, 6 mL of the loading solution was injected into the SPE cartridge, which was activated with 3 mL of chloroform. The SPE cartridge was washed with 4 mL of 1-propanol (washing solvent) to remove the matrix interferents and then fully dried under vacuum for 10 min. The dried SPE cartridge was eluted with a mixed solution of diethylether and acetic acid (98:2, *v*/*v*) to extract the analytes (MA, PA, *cis*-PA, SA, and OA). The eluate was evaporated for 20 min at 45 °C and 30 kPa under a nitrogen gas stream, using a TurboVap LV (Caliper Life Sciences, MA, USA).

### 3.3. Derivatization

**Figure 7:**
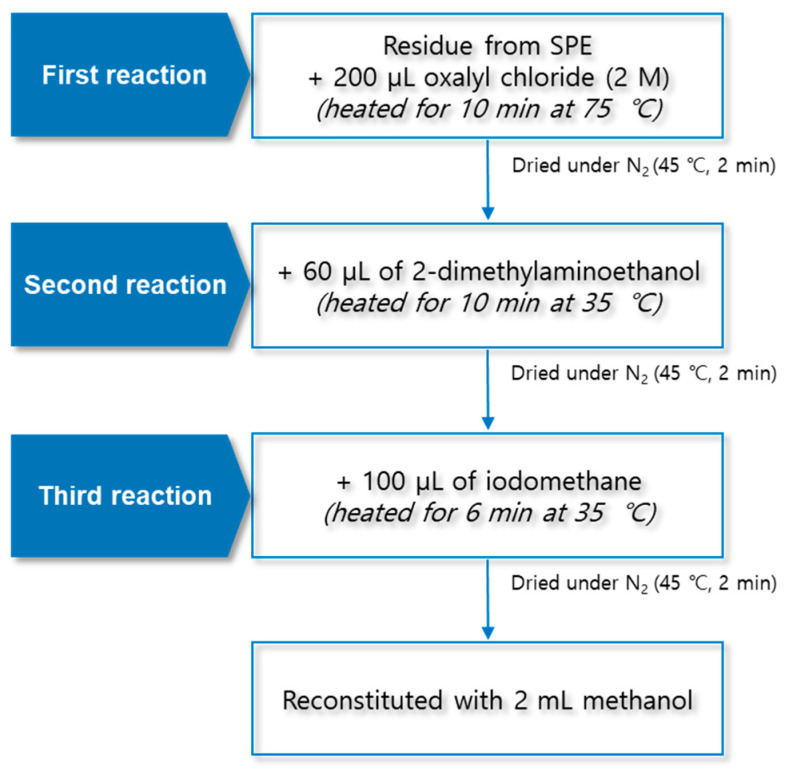
Structural diagram of trimethylaminoethyl derivatization procedure using three-step reaction.

The TMAE derivatization was carried out in a three-step reaction. First, 200 μL of 2 M oxalyl chloride was added to the final residue mentioned in Section 3.2. After the lid of the test tube was closed, the sample was reacted for 10 min at 75 °C using a block heater (QBD4, Grant Instruments, Cambridge, UK). The sample was dried for 2 min at 45 °C and 30 kPa under a nitrogen gas stream. Second, 60 µL of 2-dimethylaminoethanol was added to the residue; the reaction was carried out at 35 °C for 10 min, and the sample was dried for 2 min at 45 °C and 30 kPa under a nitrogen stream. Finally, 100 μL of iodomethane was added; the reaction was carried out at 35 °C for 6 min, and the sample was dried for 2 min at 45 °C and 30 kPa under a nitrogen gas stream. The final residue was reconstituted with 2 mL of methanol, and 10 μL was injected into the LC–MS/MS system for the analysis. The structural diagram of the TMAE derivatization procedure is shown in Figure 7.

### 3.4. LC–MS/MS Conditions

LC–MS/MS was performed using a Thermo Scientific (San Jose, CA, USA) Accela^®^ HPLC system coupled to an LCQ Fleet mass spectrometer. The target analytes were separated using a SHISEIDO CAPCELL PAK C18 MGII S3 column (2.0 × 100 mm, 3 μm particle size, Ginza, Tokyo, Japan). The mobile phase consisted of distilled water (mobile phase A) and acetonitrile containing 5 mM ammonium acetate (mobile phase B), and the flow rate was maintained at 0.3 mL/min. The gradient condition of mobile phase B was set at 40% from 0 min to 2 min, increased to 60% until 8.7 min, increased to 80% by 10 min, and maintained until 13.3 min. Finally, the condition was changed to the initial composition (i.e., 40% of mobile phase B) for 10 min to stabilize the LC system. The column was thermostated at 40 °C, while the autosampler temperature was 10 °C. The injection volume was 10 μL.

ESI was carried out in the positive mode. He gas was used as the collision gas; the capillary temperature was 275 °C, and the ion spray voltage was 6.0 kV. The precursor and product ions, capillary voltage, and collision energy for each analyte and IS are described in the Section 2 (Results and Discussion).

## 4. Conclusions

The purpose of this study was to develop an extraction method for the simultaneous analysis of five types of fatty acids (MA, PA, *cis*-PA, SA, and OA) in macroalgae, using LC–MS/MS. The introduction of TMAE derivatives to the hydroxyl functional group of the analytes significantly improved the sensitivity by overcoming the poor ionization efficiency of fatty acids in LC–MS/MS analysis. In addition, as two ionization methods (ESI and APCI) were compared, it was found that sufficient sensitivity could only be obtained using ESI. SPE was used for achieving an improved extraction efficiency and an effective sample purification. Particularly, the optimal conditions of the loading solution and washing solvent in SPE were set by comparing the recoveries under the various conditions. The developed method offers a reliable and sensitive tool for the analysis of large algae samples for their potential use in biodiesel production. This the first report on the simultaneous determination of five fatty acids in large algae, using LC–ESI–MS/MS with TMAE derivatization.

## Figures and Tables

**Table 1:** Optimized multiple reaction monitoring transitions, mass spectrometric parameters, and retention times (RTs) for analytes and internal standards derivatized using trimethylaminoethyl (TMAE).

	RT (min)	Precursor Ion (*m/z*)	Product Ion (*m/z*)	Capillary Voltage (V)	Collision Energy (%)
TMAE–Myristic Acid	9.8	314.4	255.3	22	28
TMAE–*cis*-Palmitvaccenic Acid	10.7	340.4	281.3	24	28
TMAE–Palmitic Acid	11.9	342.4	283.4	24	28
TMAE–Palmitic Acid-d_2_	11.9	344.4	285.3	20	28
TMAE–Oleic Acid	12.2	368.3	309.3	46	30
TMAE–Stearic Acid	13.5	370.4	311.4	34	32
TMAE–Stearic-d_2_ Acid	13.5	372.4	313.4	26	30

## Data Availability

The data presented in this study are available in manuscript.
